# From Sir Henry Burdett, to the Editor, "The Times"

**Published:** 1919-06-07

**Authors:** Henry C. Burdett

**Affiliations:** The Lodge, 13 Porchester Square, W. 2.


### The Central Committee's Bill.

### From Sir Henry Burdett, to the Editor, "The Times"

Sir,—It would appear from the Memorandum printed on the front page of the Central Committee's Bill, as amended by Standing Committee E (April 14, 1919), that Mr. Herbert Paterson's, and not the Hon. Sir Arthur Stanley's, letter is in error. The Memorandum sets out that the Central Committee's Bill represents "by delegation the British Medical Association" and various societies of nurses, "comprising not less than 30,000 medical practitioners and nurses." I have ascertained that the British Medical Association has 23,000 members, which leaves only 7,000 to represent the nurses whom the Central Committee thus claim to have behind them. One of the above-mentioned societies is the Fever Nurses' Association, with 2,500 nurses on its register trained in fever nursing. This Association grants "certificates of registration to fever nurses as a guarantee of efficient fever training." If these are deducted there remain 4,500 others who may or may not be fully-trained nurses.

It may be noted that Mr. Paterson nowhere speaks of fully-trained nurses as applying to the 13,000 he claims to support the Central Committee. Further, the figures given in his letter are greater than the figures set forth in the Memorandum on which Standing Committee E had to base their decision. In these circumstances it is certainly due to Parliament, the nursing profession and the public that Mr. Paterson should set forth in detail the actual number of fully-trained nurses who are supporters of the Central Committee, and the evidence of their full training in each case. The College of Nursing has always been prepared to produce this evidence, and has, I understand, in the press a register of the 14,000 fully-trained nurses, with the full history, career, and training of these nurses, who are members of the College.

There is no real substance in the other portions of Mr. Paterson's letter, for his points have been sufficiently disposed of in the Houses of Parliament and the *Times*. The claim in his final paragraph that "the College Bill is the employers' Bill" will not bear examination. Mr. Paterson, whose intimate connection with the Central Committee's Bill he fully sets forth, is, or at least was until the other day, like most members of the medical profession, an employer of nurses himself. He is surgeon-in-charge of Queen Alexandra's Hospital, Highgate.

Few there are who would maintain as a general principle that an employer is not entitled to representation, and in the present instance the contention is an attempt to camouflage the position as it in fact is. The truth is that the hospitals with training-schools, for the most part, are only employers in the sense that they employ and pay nurses to minister to the sick within their walls. The branch of their work, that of the training and education of nurses for the sick throughout the country, has been continuously maintained, mainly at the expense of these hospitals. In this way only has it been possible that the people of all classes when ill have been enabled to obtain the services of fully-qualified, up-to-date attendants, without whose aid their lot in the day of sickness would have been grievous.

I am, yours faithfully,

Henry C. Burdett.

The Lodge, 13 Porchester Square, W. 2.
June 2. (*The Times*, June 3.)

